# Diagnosis and Management of Plummer–Vinson Syndrome in a Female Ghanaian

**DOI:** 10.1155/crh/7353717

**Published:** 2026-02-25

**Authors:** Dela Fiawoo, Theophilus Tandoh, Edeghonghon Olayemi

**Affiliations:** ^1^ Department of Haematology, Korle Bu Teaching Hospital, Accra, Ghana, kbth.gov.gh; ^2^ Department of Haematology, University of Ghana Medical School, Accra, Ghana, ug.edu.gh

**Keywords:** barium swallow, case report, dysphagia, iron deficiency anaemia, iron therapy, oesophageal cancer, oesophageal webs, Plummer–Vinson syndrome

## Abstract

Plummer–Vinson syndrome (PVS), also known as Paterson–Brown Kelly syndrome, is a clinical condition that is characterized by the triad of iron deficiency anemia, dysphagia and oesophageal web. This condition is rare and is diagnosed more in middle aged to older white women. However, in some studies, younger individuals have also been found to have this condition. We present a 26‐year‐old woman with a 5‐year history of progressive difficulty in swallowing and a long history (approximately 18 years) of iron deficiency anaemia and has been on intermittent iron supplementation. A barium swallow showed narrowing in the upper third of the oesophagus, and she underwent serial dilatation of the oesophageal web stenosis and iron therapy with resultant improvement in the dysphagia. This patient was found to be pregnant 3 months postdiagnosis of PVS. The prevalence of PVS has decreased due to early diagnosis of iron deficiency and repletion of iron stores.

## 1. Introduction

The Plummer–Vinson syndrome (PVS) is named after two American Mayo clinic physicians Henry Stanley **Plummer** and Porter Paisley **Vinson** who noted patients with iron deficiency and dysphagia in the presence of suspected spasm of the upper oesophagus or abnormal angulation of the oesophagus. In the United Kingdom, PVS is also called Paterson–Brown Kelly syndrome, named after two British otolaryngologists, Adam Brown‐Kelly and Donald Ross Paterson [1]. On gross pathology, oesophageal web and oesophageal strictures are characteristic findings of PVS. On microscopic histopathological analysis, PVS shows epithelial atrophy, chronic submucosal inflammation and epithelial atypia or dysplasia in advanced cases [2, 3]. Chronic irritation of the oesophagus may predispose to an increased risk of developing oesophageal webs or strictures. PVS is considered a precancerous condition, as approximately 10% of affected individuals develop squamous cell carcinoma of the hypopharynx, oral cavity or oesophagus [4].

PVS remains underdiagnosed, particularly in resource‐limited settings, where early detection is critical to preventing progression to malignancy. A comprehensive understanding of its clinical presentation across diverse populations can facilitate timely diagnosis and intervention by clinicians worldwide.

## 2. Case Report

**FIGURE 1:**
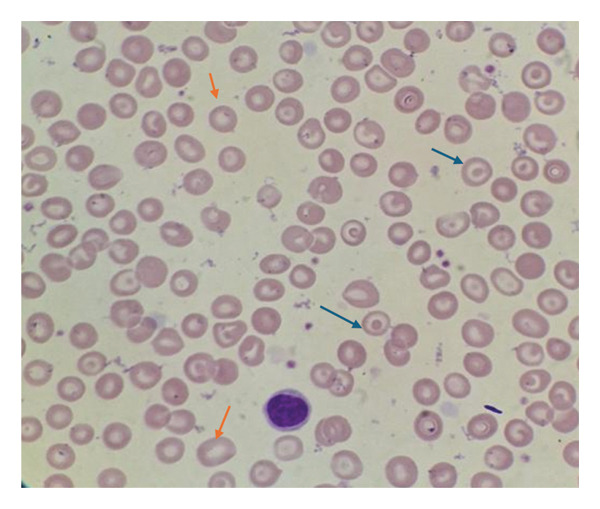
Peripheral blood smear showing anisocytosis, moderate hypochromasia (orange arrows) and a few target cells (blue arrows) (Leishman stain × 1000).

**FIGURE 2:**
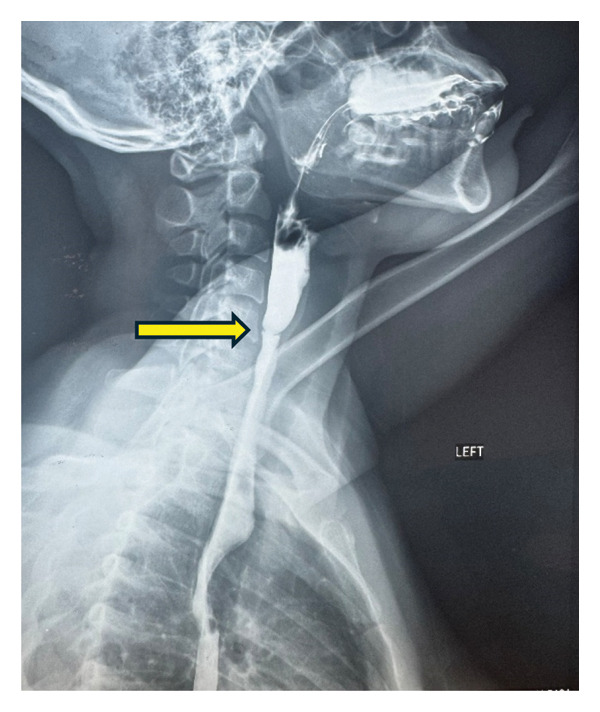
Upper oesophageal web seen on barium‐swallow oesophagography indicated by arrow.

A 26‐year‐old woman presented to the outpatient department of the Haematology unit, Korle‐Bu Teaching Hospital, Accra Ghana, in November 2024 following a referral from the ear, nose and throat (ENT) department. She presented with a history of difficulty in swallowing for 5 years and recurrent anemia since childhood. The dysphagia was progressive with difficulty swallowing solids and later liquids. She also had recurrent episodes of easy fatigue since age 7, her full blood count almost always showed haemoglobin (Hb) concentration below 10 g/dL at her hospital visits and she has been on iron supplements for most of her childhood and adult life. On physical examination, she was found to be moderately pale, and her systemic examination was normal. A direct laryngoscopy was performed by ENT surgeons which revealed normal epiglottis, valecullae and normal vocal cords mobility on both abduction and adduction. Further investigations were requested including a full blood count which showed a Hb of 11.0 g/dL (11.0–18.0 g/dL), mean cell volume of 77.7 fL (76–96 fL), mean cell Hb of 25.3 pg (27–34 pg), total white cell count 5.78 × 10^9^/L (2.5–8.5 × 10^9^/L) and platelets 261 × 10^9^/L (150 – 400 × 10^9^/L) (Table 1), blood film comment showed red cell anisocytosis, moderate hypochromasia and few target cells (Figure 1), iron studies showed serum iron 4.1 umol/L (10.7–32.2 umol/L), total iron binding capacity (TIBC) 78.01 umol/L (45.5–86.5 umol/L), ferritin 11.33 ng/mL (13–150 ng/mL) (Table 2) and a fluoroscopic barium swallow revealed a web in the upper oesophagus (Figure 2).

**TABLE 1:** Full blood count.

Parameter	Value	Reference range
Hb concentration	11.0 g/dL	11.0–18.0 g/dL
Red blood cell (RBC) count	4.2 × 10^12^/L	3.5–5.5 × 10^12^/L
Mean cell volume (MCV)	77.7 fL	76.0–96.0 fL
Mean cell haemoglobin (MCH)	25.3 pg	27.0–34.00 pg
Mean cell haemoglobin concentration (MCHC)	32.4 g/dL	31–37 g/dL
White blood cell count (WBC)	5.78 × 10^9^/L	2.5–8.5 × 10^9^/L
Platelet count	261 × 10^9^/L	150–400 × 10^9^/L

**TABLE 2:** Iron studies.

Parameter	Value	Reference range
Serum iron	4.01	10.7–32.2 umol/L
UIBC (unsaturated iron‐binding capacity)	74	27.8–63.6 umol/L
TIBC (total iron‐binding capacity)	78.01	45.5–86.5 umol/L
Ferritin	11.33	13–150 ng/mL

## 3. Blood Film Comment

The red cells showed anisocytosis with moderate hypochromasia and target cells. The leucocytes appeared adequate with normal morphology. Platelets were adequate with normal morphology.

## 4. Fluoroscopic Barium Swallow

A circumferential ring‐like narrowing in the upper third of the oesophagus with prestenotic dilatation of the proximal oesophagus was seen.

## 5. Intervention

Based on the iron deficiency anaemia, dysphagia and oesophageal webs, she was diagnosed with PVS and underwent serial dilatation of the oesophageal web stenosis with resultant improvement in the dysphagia. She was able to swallow both solids and liquids without discomfort. She was subsequently referred to the Haematology Department for further management of the anemia.

## 6. Follow‐Up and Outcomes

She was continued on ferrous sulphate 200 mg twice daily for 6 months and scheduled for 3 monthly visits. A repeat barium swallow was not done because the patient was later found to be pregnant. Her repeat iron studies 3 months post start of iron therapy did not show correction of iron deficiency (Table 3) and that could be attributed to her pregnancy.

**TABLE 3:** Iron studies pre and post therapy.

Parameter	Pretherapy	Post therapy	Reference range
Serum iron	4.01	5.25	10.7–32.2 umol/L
Transferrin		2.85	2.5–3.8 g/L
UIBC (unsaturated iron‐binding capacity)	74	59	27–63.6 umol/L
TIBC (Total iron‐binding capacity)	78.01	64.25	45.5–86.5 umol/L
Ferritin	11.33	12.37	13–150 ng/mL

## 7. Discussion

PVS is a rare disorder characterized by a triad of iron‐deficiency anaemia, dysphagia and oesophageal webs. Currently, despite worthwhile advancements in the medical field, the aetiopathogenesis of PVS remains unclear. Several mechanisms have been postulated, including iron and nutritional deficiencies, genetic predisposition and autoimmune factors [5–7] Among these, the iron deficiency theory is the most prevalent. It is thought that the depletion of iron‐dependent oxidative enzymes may produce myasthenic changes in muscles involved in the swallowing mechanism, atrophy of the oesophageal mucosa and formation of webs as epithelial complications [8]. This is supported by the improvement in dysphagia after iron therapy [9].

Some studies [8, 10] have found an association between PVS and upper gastrointestinal carcinoma. About 3%–15% of women between 15 and 50 years of age with PVS in these studies had either pharyngeal or oesophageal cancer. This suggests PVS as a risk factor to developing upper gastrointestinal cancers and thus proper follow‐up and screening are warranted in such individuals.

Some differential diagnosis of PVS include dysphagia from achalasia, reflux oesophagitis, oesophageal carcinoma, oesophageal spasm, systemic sclerosis and Zenker’s diverticulum [11]. Physical examination of patients with PVS is usually remarkable for pallor, glossitis, fatigue and weakness [12]. Laboratory findings of PVS are compatible with the presence of iron deficiency anaemia.

Diagnosis of the syndrome involves establishing the presence of iron deficiency anaemia and oesophageal webs in a patient with dysphagia. Barium swallow, video fluoroscopy or upper gastrointestinal endoscopy are diagnostic tools that can be employed in the investigation of esophageal webs. Barium oesophagogram is the best initial imaging study used for diagnosing PVS, which shows oesophageal webs. Oesophagogastroduodenoscopy (OGD) is also used to visualize oesophageal webs and dilatation in a few cases [13]. It is important to exclude other causes of dysphagia such as malignant tumors, oesophageal burns and heterotopic gastric mucosa [14, 15].

As PVS is rare, descriptions of the disease and management have been gathered from small case series, retrospective and prospective studies, with the largest including only 30 patients diagnosed with PVS [16]. The mainstay of treatment for PVS is aimed at correcting iron deficiency anaemia. Management options include iron therapy and serial dilations of the oesophageal webs. However, Tahara et al. [17] demonstrated that iron supplementation alone can resolve the dysphagia in some patients. Mechanical dilatation using oesophageal bougies or balloon dilators are equally effective methods of relieving oesophageal webs [18]. In one study, a cuffed endotracheal tube was used in the oesophageal dilatation [19].

In this case, the patient’s repeat iron profile after 3 months of therapy was found to show low serum ferritin, and this could be explained by her current pregnancy. In pregnancy, serum ferritin concentrations gradually decrease to reach the lowest concentrations in the third trimester [20]. In liaison with her obstetrician, close follow‐up of her iron status will be done to ensure she has good iron stores and prevent a relapse of the oesophageal web.

## 8. Patient Perspective

Patient expresses gratitude for her overall care. She is happy that her diagnosis was made, and she got instant relief after her procedure. She, however, expressed that her diagnosis was a little delayed because some doctors with the ENT team doubted the diagnosis initially and it was probably due to how rare the condition is.

## 9. Conclusion

PVS, although uncommon, is a relatively easily treatable condition. Differential diagnosis of dysphagia should always be excluded in the management of PVS. Regular follow‐up is also essential in the surveillance of upper gastrointestinal carcinoma since PVS has been demonstrated to be a risk factor. There is, however, no consensus on the surveillance guidelines for upper endoscopy for PVS, thus local guidelines in consultation with the otolaryngologist and gastroenterologist will be recommended.

This case highlights the importance of early diagnosis and management of PVS to prevent potential complications, such as increased risk of upper gastrointestinal malignancies. Increased awareness among clinicians is essential for appropriate intervention and improved patient outcomes, especially in resource‐limited settings.

## Funding

No funding was received for this manuscript.

## Conflicts of Interest

The authors declare no conflicts of interest.

## Data Availability

The data that support the findings of this study are available from the corresponding author upon reasonable request.
